# Federal Interventions Targeting Social Isolation and Loneliness: An Exploratory Review

**DOI:** 10.1093/geroni/igab046.1929

**Published:** 2021-12-17

**Authors:** Lauren Palmer, Jennifer Howard, Helena Voltmer, Abigail Ferrell, Natalie Mulmule, Abbie Levinson, Sarita Karon

**Affiliations:** 1 RTI International, RTI International, Massachusetts, United States; 2 RTI International, Durham, North Carolina, United States; 3 RTI International, Waltham, Massachusetts, United States; 4 Lake Erie College of Osteopathic Medicine, Erie, Pennsylvania, United States; 5 RTI International, RTI International, North Carolina, United States

## Abstract

Even prior to the COVID-19 Public Health and Medical Emergency, the experiences of chronic social isolation and loneliness (SIL) were growing among older adults. Countries began increasing national visibility for these issues and implementing programs and services focused on addressing them. In the United States (US), however, little is known about successful national interventions or their effectiveness in tackling SIL among older Americans. We conducted a rapid review of the peer-reviewed and grey literature from 2009-2019, focusing on existing federal programs, health systems, and health care models in the US that address SIL among older adults. Of the 110 articles identified, 36 met the inclusion criteria and were synthesized. Our review found few federal interventions that directly address SIL; several may be addressing SIL as an auxiliary outcome to addressing social determinants of health, such as group exercise, transportation support, or food insecurity. While these interventions may provide a promising opportunity, implementation and evaluation challenges were identified. Thus, federal and state agencies face significant obstacles to understanding the impact of existing interventions and their effectiveness in addressing SIL, hampering progress toward large scale implementation. As SIL receives increasing attention, we add another voice to existing literature that indicates significant heterogeneity among existing programs; we found that few evidence-based, scalable federal initiatives exist in the US that target SIL. Without resources from federal and state agencies, the ability of health entities, community-based organizations, and direct care providers to implement effective interventions is significantly diminished.

